# COVID-19 associated mucormycosis in Assiut University Hospitals: a multidisciplinary dilemma

**DOI:** 10.1038/s41598-022-13443-3

**Published:** 2022-06-21

**Authors:** Sahar Farghly Youssif, Marwa M. Abdelrady, Ahmed Atef Thabet, Mohamed A. Abdelhamed, Mohamed Omar A. Gad, Ahmed Mohmmed Abu-Elfatth, Ghada Mohamed Saied, Islam Goda, Abdelazeem M. Algammal, Gaber El-Saber Batiha, Nessren M. Abd el-Rady, Helal F. Hetta, Soheir M. Kasem

**Affiliations:** 1 Department of Chest Disease and Tuberculosis, Faculty of Medicine, Assuit University, Assiut, 71515 Egypt; 2 Department of Anesthesia and Critical Care, Faculty of Medicine, Assuit University, Assiut, 71515 Egypt; 3 Department of Internal Medicine and Critical Care, Faculty of Medicine, Assuit University, Assiut, 71515 Egypt; 4 Department of Neurology and Psychiatry, Faculty of Medicine, Assuit University, Assiut, 71515 Egypt; 5 Department of Otorhinolaryngology, Faculty of Medicine, Assuit University, Assiut, 71515 Egypt; 6 Department of Tropical Medicine and Gastroenterology, Faculty of Medicine, Assuit University, Assiut, 71515 Egypt; 7 Department of Clinical Pathology Department, Faculty of Medicine, Assuit University, Assiut, 71515 Egypt; 8 Department of Ophthalmology Department, Faculty of Medicine, Assuit University, Assiut, 71515 Egypt; 9 Department of Bacteriology, Immunology and Mycology, Faculty of Veterinary Medicine, Suez Canal University, Ismailia, 41522 Egypt; 10 Department of Pharmacology and Therapeutics, Faculty of Veterinary Medicines, Damanhour University, Damanhour, 22511 Egypt; 11 Medical Physiology Department, Faculty of Medicine, Assiut University, Assiut, 71515 Egypt; 12 Department of Medical Microbiology and Immunology, Faculty of Medicine, Assuit University, Assiut, 71515 Egypt

**Keywords:** Microbiology, Diseases

## Abstract

Mucormycosis is a life-threatening opportunistic angioinvasive fungal infection. We aimed to describe the frequency, presentations, predictors, and in-hospital outcome of mucormycosis patients in the scope of CoronaVirusDisease-19 (COVID-19) during the third viral pandemic wave. This cross-sectional retrospective study included all patients who fulfilled the criteria of mucormycosis with concurrent confirmed covid19 infection admitted to Assuit University Hospital between March 2021 and July 2021. Overall, 433 patients with definite covid-19 infection, of which 33 (7.63%) participants were infected with mucormycosis. Mucormycosis was predominantly seen in males (21 vs. 12; p = 0.01). Diabetes mellitus (35% vs. 63.6%; p < 0.001), hypertension (2% vs.45.5%; p 0.04), and Smoking (26.5% vs. 54.5%; p < 0.001) were all significantly higher in mucormycosis patients. Inflammatory markers, especially E.S.R., were significantly higher in those with mucormycosis (p < 0.001). The dose of steroid intake was significantly higher among patients with mucormycosis (160 mg vs. 40 mg; p < 0.001). Except for only three patients alive by residual infection, 30 patients died. The majority (62%) of patients without mucormycosis were alive. Male sex; Steroid misuse; D.M.; Sustained inflammation; Severe infection were significant risk factors for mucormycosis by univariate analysis; however, D.M.; smoking and raised E.S.R. were predictors for attaining mucormycosis by multivariate analysis.

## Introduction

The coronavirus disease 2019 (COVID-19) pandemic has been related to aggressive bacterial and fungal diseases^1^. Mucormycosis is a fungal infection caused by Mucorales fungi such as Rhizopus, Mucor, Rhizomucor, Cunninghamella, and Lichtheimia^2^. Phagocytes are the primary host defense against mucormycosis^3^.

Mucormycosis infection is characterized by extensive angio-invasion, culminating in arterial thrombosis and tissue destruction^4^. The clinical presentation of mucormycosis is variable because it spreads rapidly into the body systems; it affects the paranasal sinuses, then orbits, central nervous system, lungs, gastrointestinal system, kidneys, and skin. Manifestations of mucormycosis usually depend on: (a) the route of entry of fungal spores into the body, mainly by inhalation or ingestion or direct skin injection; (b) the comorbid diseases of the infected patients^2^.

Mucormycosis is more common in immunocompromised individuals. Diabetic ketoacidosis and steroids are the most common risk factors for mucormycosis infection. And complications such as ocular and brain involvement^5^. As corticosteroid treatment impairs macrophages' ability to prevent mucor fungi spores from germinating^4^. Moreover, high glucose due to diabetes, new-onset hyperglycemia or steroid-induced hyperglycemia is considered an ideal low oxygen environment (hypoxia).

There are many other risk factors for mucormycosis like increased ferritin, acidic medium (metabolic acidosis, diabetic ketoacidosis [DKA]) and prolonged hospitalization with or without mechanical ventilators. Also, mucormycosis can occur due to decreased phagocytic activity of white blood cells (WBC) resulting from immune suppression caused by coronavirus 19 related severe acute respiratory syndrome (SARS-CoV-2)^6^. Therefore, mucormycosis in those patients suffering or recovering from COVID-19 infection increased worldwide because COVID-19 infection provides a suitable environment for mucormycosis^7^.

Even with appropriate treatment, the rhino-orbital infection caused by the Mucorales fungus has a terrible prognosis, with up to 50% mortality^8^. Mucormycosis is treated by early diagnosis and controlling risk factors, then by proper surgical debridement and administration of the suitable antifungal medication. Liposomal amphotericin B (LAmB) is an efficient antifungal drug for treating mucormycosis with a minimum of 5 mg/kg. However, posaconazole and voconazole are recommended for severe refractory cases^9^.

Given that the current pandemic is still a significant public health concern worldwide, patients with COVID-19 should be mindful of mucormycosis, as the two illnesses can cause severe morbidity and mortality when combined. The study's objective was to describe the frequency, presentations, predictors, and in-hospital outcomes of mucormycosis patients in the context of COVID-19.

## Materials and methods

### Ethical statement and informed consent

We conducted a retrospective cross-sectional observational study at Assuit University Hospital's tertiary-care center. The study was conducted according to the guidelines of the Declaration of Helsinki and approved by the Institutional Review Board of Assiut University College of Medicine (IRB.17300662), and the study protocol has been registered on clinical trials.gov (NCT05074043).

Informed consent was obtained from all subjects involved in the study. All patients provided informed consent after being informed that we could utilize their clinical and radiological data and face photos for research purposes and publication of identifying information/images in an online open-access publication.

### Study participants

In this retrospective cross-sectional study, the clinical-record analysis included all patients who fulfilled the criteria of mucormycosis with concurrent confirmed covid19 infection who were admitted to Assuit University Hospital between March 2021 and July 2021 during the third pandemic wave to describe the frequency; presentations; predictors; the in-hospital outcome proved cases of mucormycosis infection in the context of COVID-19 diseases. With the cooperation of internists, intensivists, neurologists, otolaryngologists; ophthalmologists; and pathologists who help in confirmation of mucormycosis diagnosis by Clinical, radiological, and histopathological assessment.COVID-19 infection in the patients' records was evidence of a positive reverse transcription-polymerase chain reaction (RT-PCR) test for SARS-CoV-2. The fulfilled criteria for diagnosis of mucormycosis were based on the global guideline for diagnosing and managing mucormycosis published by the European Confederation of Medical Mycology in cooperation with the Mycoses Study Group Education and Research Consortium^10^. Non-confirmed COVD-19 patients by polymerase chain reaction (PCR) or suspected fungal infection were excluded from the study.

### Assessment parameters

#### Clinical and demographics

Clinical records included patient demographics, clinical data, including age, weight, gender, body mass index (B.M.I.), and COVID-19 severity, as defined by World Health Organization (WHO) guidelines: mild, moderate, and severe was recorded^11^. Also, clinical records includedMucormycosissympyoms and signs, corticosteroids use, dose and duration, and other treatment modalities (antibiotics, antifungal drugs and any adjunct surgery performed for mucormycosis). In-hospital outcomes and comorbidities like the presence or absence of diabetes and its duration are also covered in the recorded data. Other comorbidities, i.e., hypertension (Cardiac, renal disease, chronic hepatic diseases. Cardiac diseases according to WHO classification^12^. Renal disease, whether acute kidney injury (A.K.I.)^13^, which is defined as any of the following: increase in serum creatinine ≥ 0.3 mg/dl within 48 h or increased serum creatinine to ≥ 1.5 times baseline within 7 days or urine volume < 0.5 ml/kg/h for 6 h) or chronic kidney disease (CKD) (Kidney damage for ≥ 3 months, as defined by structural or functional abnormalities of the kidney)^14^.

### Laboratory; radiological and histopathological assessment

#### Laboratory

d-dimer, blood glucose, C-reactive protein, ferritin, creatinine; Liver function; C.B.C., electrolyte. COVID-19 was diagnosed using real-time polymerase chain reaction (RT-PCR) assays from nasopharyngeal or oro-pharyngeal swabs and C.T. chest scan (HRCT). Mucormycosis was confirmed through specific histological characteristics in biopsy specimens based on the global guideline for the diagnosis and management of mucormycosis^10^.

#### Radiology

For patients, a computerized tomography (C.T.) scan of the orbit, paranasal sinuses, and lung were done as the initial imaging examination to determine the clinical and histological types of mucormycosis, Gadolinium-enhanced magnetic resonance imaging (M.R.I.) of the orbits, brain and paranasal sinuses was also conducted for individuals with symptoms that included M.R.V.

#### Histopathology

Nasal mucosal tissue biopsies were obtained from the E.N.T. Department, Assiut University Hospital. Biopsies were immediately fixed in 10% formalin solution and processed by automated tissue processing till paraffin embedding, and then biopsies were serially cut into 5 µm thick sections on a glass slide. The slide was stained with hematoxylin and eosin (H&E) for microscopic evaluation using an Olympus CX41 microscope. If H&E stained sections suspected fungal bodies, other sections were prepared and stained with GomoriMethenamine Silver (G.M.S.) stain to confirm and detect the precise morphology of the fungal bodies.

#### Surgical procedures

Multiple sessions of transnasal endoscopic debridement of necrotic tissue from the sinonasal tract extended to the orbit and skull base in affected patients.

#### Outcomes

The primary endpoint of our study was to detect the predictors of mucormycosis in COVID-19 patients. Secondary endpoints included the frequency and in-hospital mortality of C.A.M.

#### Sample size

Our study was retrospectively enrolled all COVID 19 patients in a specific period to estimate the frequency of C.A.M. (Covid associated mucormycosis).

### Statistical analysis

Data was collected and analyzed using SPSS (Statistical Package for the Social Science, version 20, I.B.M., and Armonk, New York). The Shapiro test was used to determine compliance of the data to normal distribution. Quantitative data with normal distribution were expressed as mean ± standard deviation (S.D.) and compared with the Student t-test. Quantitative data with abnormal distribution expressed as median (minimum–maximum) and compared by the Mann–Whitney test was used.

Nominal data were given as a number (n) and percentage (%). *Chi*^2^ test was implemented on such data. Predictors of mucormycosis among patients with COVID-19 infection were determined by logistic regression analysis. The confidence level was kept at 95%, and hence, the P-value was considered significant if < 0.05.

### Institutional Review Board statement

The study was conducted according to the guidelines of the Declaration of Helsinki, and approved by the Institutional Review Board of Assiut University College of Medicine (IRB.17300662) and the study protocol have been registered in Clinicaltrials.gov (NCT050740043).

### Informed consent statement

Informed consent was obtained from all subjects involved in the study. All patients provided informed consent after being informed that we could utilize their clinical and radiological data and face photos for research purposes and publication of identifying information/images in an online open-access publication.

## Results

**Figure 1:**
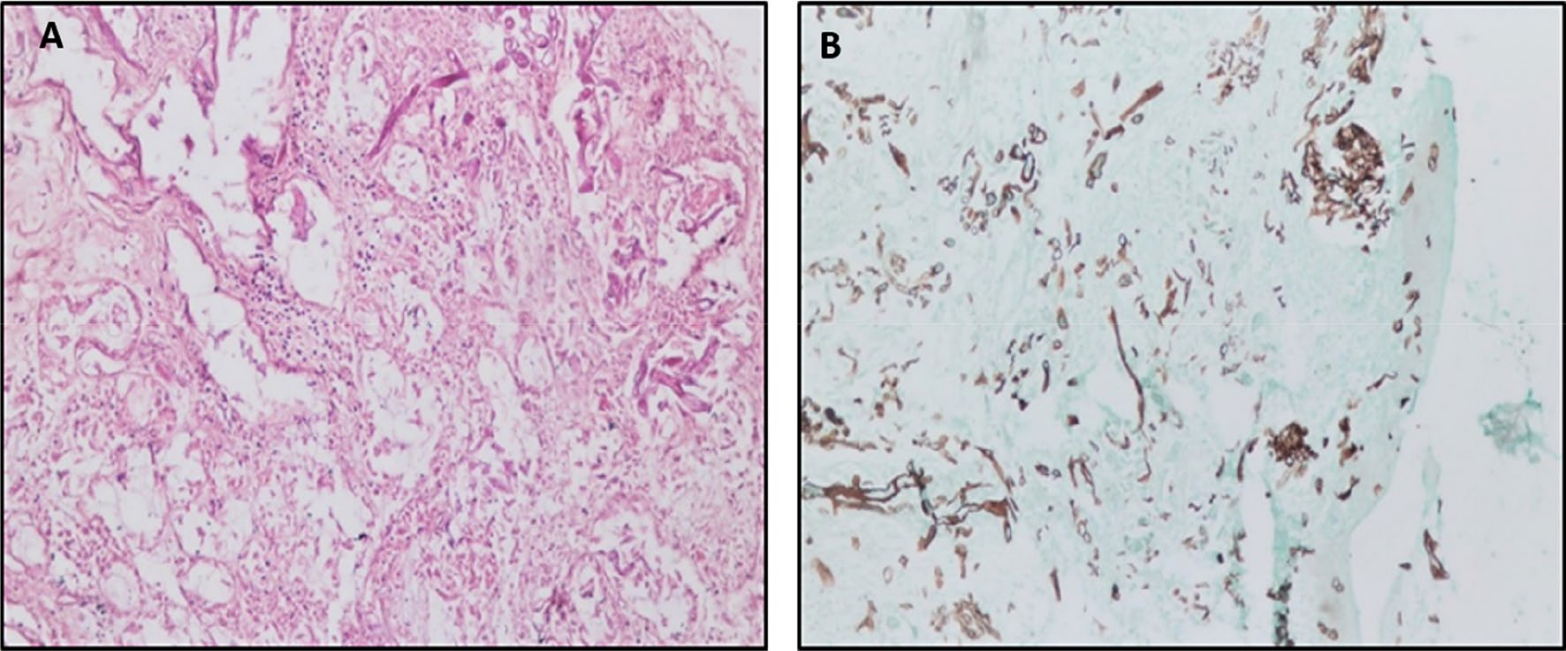
Histopathology of nasal mucormycosis. (**A**) A photomicrograph of a section of nasal mucormycosis that shows obvious necrosis of nasal mucosal glands and mononuclear inflammatory infiltrate (H&E stain, ×200). (**B**) GomoriMethenamine Silver fungal stained section of the same case in this figure highlights branched, thick fungal bodies were invading the nasal mucosal tissue (G.M.S. stain, ×200).

**Figure 2:**
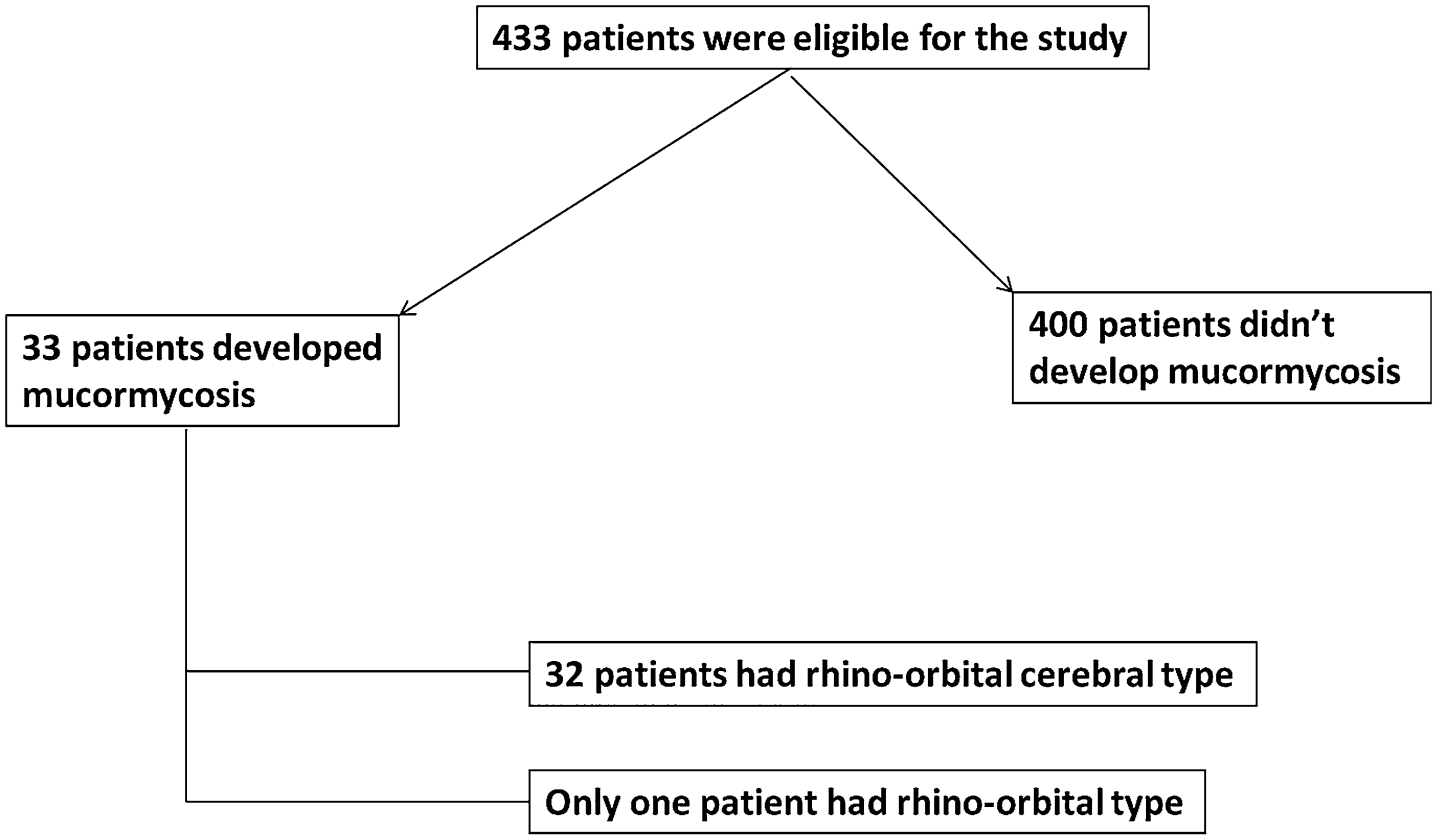
Flow chart of the studied population.

The current study enrolled 433 patients with confirmed COVID-19 infection; 33 (7.6%) patients were infected with mucormycosis based on histopathology results (Fig. 1A,B). So, the enrolled patients were subdivided based on the development of mucormycosis into; patients who were infected with mucormycosis, known as the study group (n = 33), and those who didn't infect with mucormycosis known as the control group (n = 400) (Fig. 2).

### Baseline data of enrolled patients

Baseline line data showed that both groups had a significant difference as regard sex distribution with male predominance among patients with mucormycosis (21 (63.6%) vs. 175 (43.8%); p = 0.02). Also, patients with mucormycosis had significantly higher frequency of smoking (18 (54.5%) vs. 106 (26.5%); *p* < 0.001), hypertension (15 (45.5%) vs. 116 (29%); p = 0.04) and diabetes mellitus (21 (63.6%) vs. 140 (35%)). Other data are summarized in Table 1.

**Table 1:** Baseline data of enrolled patients:

	COVID 19 (n = 400)	CAM (n = 33)	P value
Age (years)	53 (18–92)	58 (18–94)	0.19
**Sex**			
Male	175 (43.8%)	21 (63.6%)	**0.02**
Female	225 (56.3%)	12 (36.4%)	
**Occupation**			
None	295 (73.8%)	25 (75.8%)	0.49
Employee	105 (26.3%)	8 (24.2%)	
Current smoking	106 (26.5%)	18 (54.5%)	** < 0.001**
Dyspnea	400 (100%)	33 (100%)	–
Cough	400 (100%)	21 (63.6%)	** < 0.001**
Diabetes mellitus	140 (35%)	21 (63.6%)	** < 0.001**
Hypertension	116 (29%)	15 (45.5%)	**0.04**
Renal disease	28 (7%)	3 (9.10%)	0.42
Liver disease	28 (7%)	4 (12.12%)	0.21
Cardiac disease	17 (4.3%)	3 (9.1%)	0.18
Duration of symptoms (days)	14.46 ± 1.78	14.82 ± 2	0.27

Data expressed as frequency (percentage), mean (SD). *P* value was significant if < 0.05. Nominal data was compared by Chi-square test while age was compared by Mann Whitney test while duration of symptoms was compared by Student t test.

CAM: covid associated mucormycosis.

Significant values are given in bold.

### Clinical, laboratory and radiological data among enrolled patients

Patients with mucormycosis had significantly higher random blood sugar and lower leucocytes and hemoglobin level. Also, the erythrocyte sedimentation rate was significantly higher among those patients with mucormycosis (54.5% vs. 12%; *p* < 0.001). Based on chest radiology, all patients with mucormycosis had ground-glass opacity (G.G.O.). Patients with mucormycosis had a more severe disease where 48.5% and 30.3% of the severe and life-threatening disease, meanwhile 50% of those without mucormycosis had moderate disease severity.

Both groups had insignificant differences as regard SOFA score (p = 0.47).

The SOFA (Sequential Organ Failure Assessment) score can determine the level of organ dysfunction and mortality risk in I.C.U. (Intensive Care Unit) patients. based on six scores, one for each respiratory, cardiovascular, hepatic, coagulation, renal and neurological system, each scored from 0 to 4 with an increasing score reflecting worsening organ dysfunction^15^. Other data are summarized in Table 2.

**Table 2:** Clinical, laboratory and radiological data among enrolled patients.

	COVID-19 only (n = 400)	CAM (n = 33)	*P* value
Heart rate (b/m)	86.91 ± 8.25	85.67 ± 8.82	0.41
Temperature (ºC)	38.29 ± 0.68	38.27 ± 0.69	0.95
Respiratory rate (c/m)	28.75 ± 1.67	29.06 ± 2.26	0.32
Baseline hypoxemia	328 (82%)	28 (84.8%)	0.44
RBS (mg/dl)	140 (90–298)	210 (121–240)	** < 0.001**
Leucocytes (10^3^/µl)	13 (1–34)	10 (6–16)	** < 0.001**
Lymphocytes (10^3^/µl)	1.30 (0.20–4)	1.2 (0.30–3)	0.97
RDW (%)	14.83 ± 2.81	15.73 ± 3.33	0.08
Hemoglobin (mg/dl)	13.37 ± 1.39	12.11 ± 1.54	** < 0.001**
Platelets (10^3^/µl)	205.87 ± 60.56	221.39 ± 76.26	0.16
Raised ESR	48 (12%)	18 (54.5%)	** < 0.001**
CRP (mg/dl)	32 (0.50–119)	24 (4–180)	0.27
d-dimer (ng/dl)	0.60 (0.03–76)	0.50 (0.10–95)	0.60
SOFA score	5 (2–17)	5 (2–18)	0.39
**Chest radiology**^**a**^			
Consolidation	116 (29%)	0	** < 0.001**
GGO	160 (40%)	33 (100%)	
GGO and consolidation	124 (31%)	0	
**Severity of COVID-19**			
Moderate	200 (50%)	7 (21.2%)	** < 0.001**
Severe	160 (40%)	16 (48.5%)	
Life threatening	40 (10%)	10 (30.3%)	

Data expressed as frequency (percentage), mean (SD), median (range) as appropriate. *P* value was significant if < 0.05.

Nominal data was compared by Chi-square test while all continuous data was compared by Student t test with exception of RBS, leucocytes, lymphocytes, CRP, D-dimer and SOFA were compared Mann Whitney test.

RBS: random blood sugar; RDW: red cell distribution width; ESR: erythrocyte sedimentation rate; CRP: C-reactive protein; GGO: ground glass opacity; SOFA: sequential organ failure assessment; COVID-19: coronavirus disease 2019. RBS: random blood sugar.

Significant values are given in bold.

^a^Included chest computed tomography and plain radiograph.

### Therapy among enrolled patients

There were significant differences between both groups regarding the use of cephalosporin, Teicoplanin, and Carbapenems, where the use of those agents was significantly higher among patients without mucormycosis. The dose of steroid intake was significantly higher among patients with mucormycosis (160 (160–200) vs. 40 (40–200) (mg); p < 0.001) Table 3.

**Table 3:** Therapy among enrolled patients.

	COVID-19 only (n = 400)	CAM (n = 33)	*P* value
Cephalosporin	192 (48%)	9 (27.3%)	**0.01**
Quinolones	204 (51%)	16 (48.5%)	0.46
Averozolid	48 (12%)	6 (18.2%)	0.21
Teicoplanin	40 (10%)	0	**0.03**
Carbapenems	144 (36%)	3 (9.1%)	** < 0.001**
Other antibiotics	8 (2%)	0	0.52
Steroid dose (mg)	40 (40–200)	160 (160–200)	** < 0.001**

Data expressed as frequency (percentage), median (range) as appropriate. *P* value was significant if < 0.05. Nominal data was compared by Chi-square test while continuous data was compared by Mann Whitney test.

CAM: covid associated mucormycosis.

Significant values are given in bold.

Antifungal treatment (Amphotericin B) was given to only ten mucormycosis patients; seven patients died before the completion of their doses, and three were improved with residual infection for further surgical intervention and antifungal treatment.

### Presentation among patients with mucormycosis

Except for one patient presented by rhino orbital affection.; the remaining patients presented by rhino-orbital—cerebral type. The majority of patients presented with headache and nerve palsy (mostly ocular nerves; 3rd, 4th, 6th, 5th) (81.81%); periorbital swelling and proptosis in 78.78%, which consecutively leads to diminution and loss of vision in 75.75%; hemiparesis was the least recorded neurological presentation in 9.09% (Table 4).

**Table 4:** Presentation and neurological manifestations among patients with mucormycosis.

	N = 33
**Presentation**	
Rhino orbital	1 (3.03%)
Rhino orbital cerebral	32 (96.96%)
**Neurological manifestions**	
Headache	28 (84.84%)
Nerve palsy (3rd, 4th, 5th, 6th)	27 (81.81%)
Periorbital swelling/proptosis	26 (78.78%)
Hemiparesis	3 (9.09%)
Diminution/loss of vision	25 (75.75%)

Data expressed as frequency (percentage).

### Predictors of mucormycosis among patients with COVID-19 infection

Based on the current study, the predictors for the development of mucormycosis among patients with COVID-19 diabetes mellitus with an odd's ratio (OR) was 13.99 (95% confidence interval (CI) = 1.96–29.79), and current smoking with OR was 3.49 (95% CI = 3.13–6.33) as well as raised E.S.R. with OR was 2.69 (95% CI = 1.71–4.03) (Table 5).

**Table 5:** Predictors of mucormycosis among patients with COVID-19 infection.

	Univarite regrssion analysis			Multivarite regrssion analysis		
	OR	95% CI	*P* value	OR	95% CI	*P* value
Male sex	1.11	0.98–1.76	0.03	1.17	0.22–6.30	0.84
Diabetes mellitus	11.34	1.44–28.87	< 0.001	13.99	1.96–29.79	** < 0.001**
Hypertesnion	1.04	1.01–2.22	0.04	1.08	0.16–7.22	0.93
Current smoking	2.67	2.01–4.55	0.01	3.49	3.13–6.33	** < 0.001**
GGO	1.13	1.10–3.09	0.04	1.23	0.78–3.01	0.99
Severe infection	1.41	1.22–4.32	0.03	1.81	0.24–13.31	0.55
Anaemia	1.06	1.01–2.92	0.03	1.01	0.22–2.50	0.99
Leucouytosis	0.89	0.22–0.99	0.04	0.56	0.10–3.22	0.52
Raised ESR	1.90	1.14–2.30	0.01	2.69	1.71–4.03	**0.01**
Steroid dose	1.11	1.01–2.30	0.03	1.09	0.89–3.01	0.99

COVID-19: coronavirus disease 2019; CI: confidence interval; ESR: erythrocyte sedimentation rate; OR: odd’s ratio; GGO: ground glass opacity.

Significant values are given in bold.

*P* value was significant if < 0.05.

### Outcome among enrolled patients

Except for only three patients alive by residual infection (one patient completely cured after antifungal and surgical debridement; 2^nd^ 3^rd^ by ptosis and ophthalmoplegia on treatment with diminution of vision); all other patients with mucormycosis (30 patients) were deteriorated and died. In contrast, the majority (63%) of patients without mucormycosis improved and were alive (Table 6).

**Table 6:** Outcome among enrolled patients.

	COVID 19-only (n = 400)	CAM (n = 33)	P value
**Outcome**			
Alive	252 (63%)	3 (9.1%)	< 0.001
Died	148 (37%)	30 (90.9%)	

Data expressed as frequency (percentage). *P* value was significant if < 0.05. Data was compared by Chi-square test.

### Specific case scenarios of the enrolled patients (Figs. 3 and 4)

**Figure 3:**
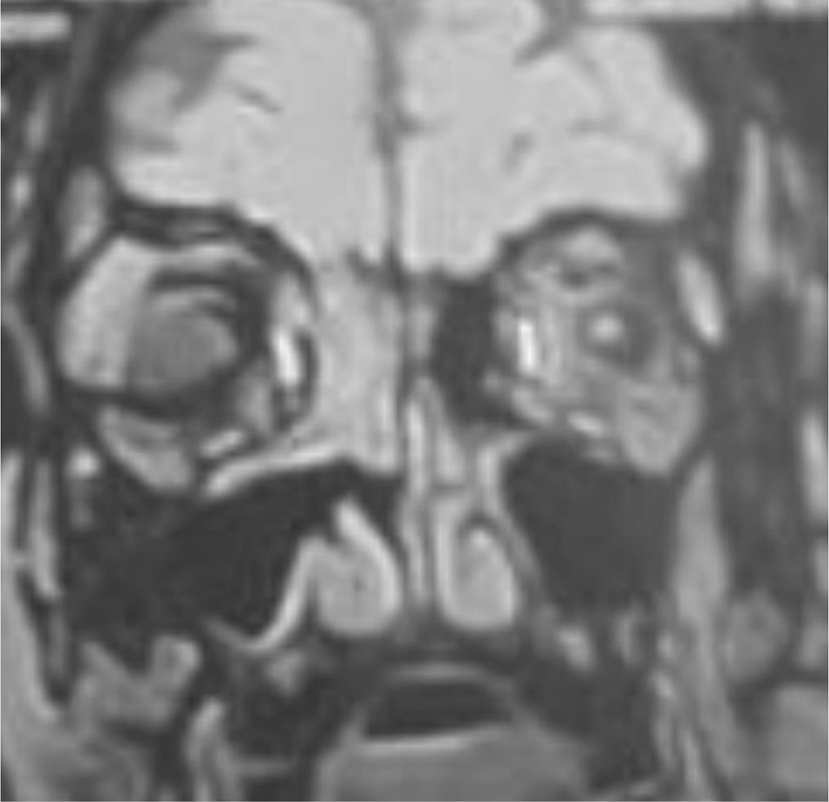
Female patient with C.A.M. presented by Rt. Maxillary and ethmoid sinuses involvement.

**Figure 4:**
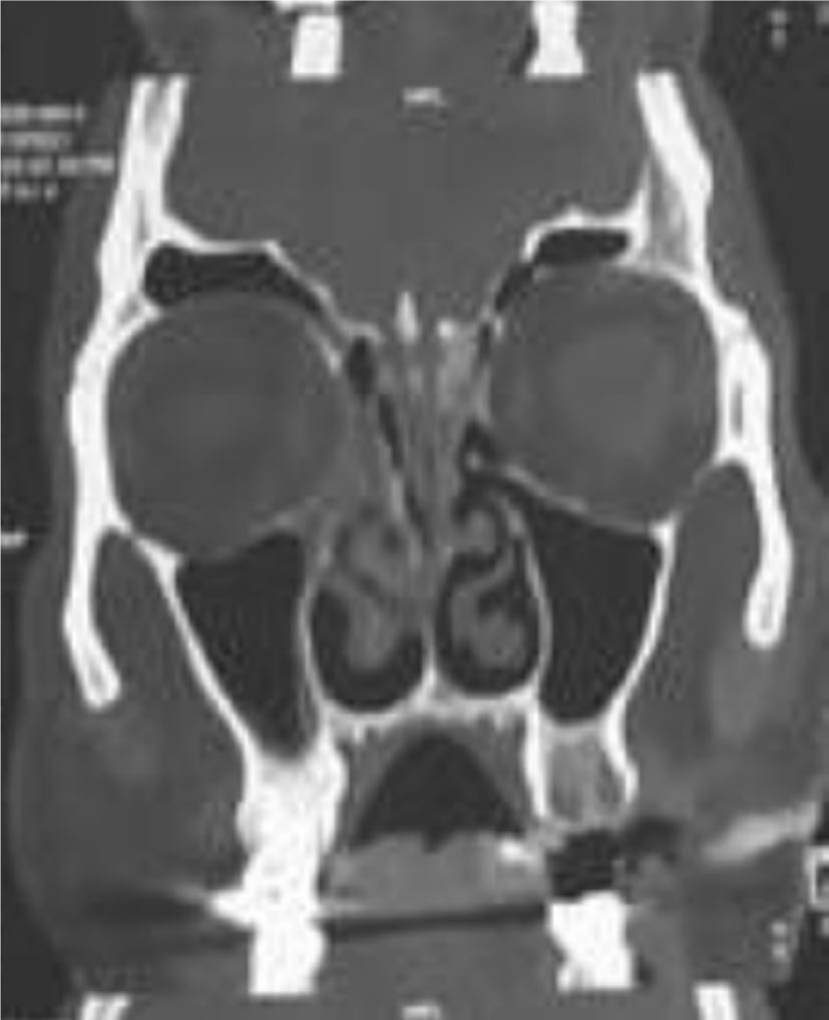
A male patient presented with bilateral rhino-fascial -cerebral CAM.

*Case (1)* Female patient;35y old; housewife from; Egypt; diabetic, presented by Fever; cough; respiratory failure diagnosed as severe COVID-19 by PCR; C.T. chest; admitted to I.C.U. for 1 month; received high doses of steroids injection (both methylprednisolone; dexamethasone) for 1 month then oral prednisolone for 1 month(60-40-30 mg); presented to neurology clinic by rt.3rd nerve palsy. Brain M.R.I. M.R.V. (Magnetic Resonance Venography) and C.T.; M.R.I. sinus were done with evidence of opacities on the rt side. E.N.T. surgical intervention was done with biopsy from maxillary and ethmoid sinuses mucosa showed invasive granulomatous fungal sinusitis mucormycosis. After surgical debridement and antifungal treatment, partial improvement then, she developed progressive ptosis and ophthalmoplegia, which need another surgical intervention and till now, she is still under repeated debridement and antifungal with progressive ophthalmoplegia and diminution of vision (Fig. 3).

*Case (2)* Male patient 62 y old; from Assiut; Egypt; heavy smoker; uncontrolled D.M., CKD grade 4 (estimated G.F.R. = 13.9 ml/min/1.73 m^2^); presented by bilateral rhino-facial mucormycosis and bilateral 1st;3rd,4th and 6th C.N. palsy. Diagnosed COVID-19 (by C.T. chest and PCR) during preparation for E.N.T. (Ear, Nose, and Throat) intervention. Brain M.R.I. M.R.A. C.T. sinuses with biopsy and histopathology were done to confirm the diagnosis of rhino orbital cerebral black fungus complicated by right cavernous sinus thrombosis and complete internal carotid artery occlusion. The patient showed rapid deterioration with sepsis and multi-organ failure and died (Fig. 4).

## Discussion

The COVID-19 pandemic has been related to aggressive bacterial and fungal infections.The current analysis comprised 433 cases, including 33 cases of COVID-19 associated Mucormycosis (C.A.M.) (8.25%) reported between March 2021 and July 2021 during the third pandemic wave providing an updated summary of this rare clinical entity. This percentage matched with the most published articles in this context; Rawson et al. found that 8% of corona virus-positive or recovered participants had secondary fungal or bacterial infections during hospitalization, despite frequent administration of broad-spectrum antibiotics and steroids^16^. There was male predominance with a history of diabetes, hypertension, smoking, and glucocorticoid medication for COVID-19 control. C.A.M. patients had a high mortality rate, although adjunct surgery for mucormycosis and antifungal treatment had been given for C.A.M. cases as recorded in clinical data for better clinical results.

Old age men, smokers, diabetics, hypertensive patients, and cardiac patients have a higher risk of developing mucormycosis, which could be attributed to a change in their innate immunity, and catching a more severe and persistent COVID-19 infection and using steroids concurrently. Most of our mucormycosis patients had severe COVID-19 illness, and they all had bilateral lung affection.

Aside from the catastrophic pulmonary consequences of smoking, it is also a powerful predictor of black fungus since it can be a significant underlying cause of fungi's oral proliferation^17^.

Mucormycosis is associated with diabetes mellitus, a substantial risk factor^18^. The majority of the patients had diabetes mellitus (63.6%). According to existing evidence, people lacking phagocytes or a reduced phagocytic activity may be more prone to mucormycosis^19^. Neutrophils are required to stop fungal spores from spreading. In a normal host, Mucorales are also eliminated by mononuclear and polymorphonuclear phagocytes, which create reactive oxygen species and cationic peptides called defensins^3^. Hyperglycemia causes phagocyte malfunction, poor chemotaxis, and inefficient intracellular killing by oxidative and non-oxidative processes, as shown in patients with uncontrolled diabetes mellitus^20^.

Smoking is a powerful predictor of black fungus since it can operate as a crucial underlying factor in the oral proliferation of fungi, as reported by Ali et al.^17^, in addition to serious pulmonary problems.

Like the ones found in COVID-19 patients, excess cytokines can aggravate insulin resistance^21^. Insulin resistance is induced by interleukin-6 (IL-6), affecting insulin receptor and substrate-1 phosphorylation, common in COVID-19 patients^22^. One-third of those with mild COVID-19 had elevated IL-6 levels, which may contribute to dysglycemia in these patients^23^. Finally, COVID-19 treatments such as glucocorticoids, lopinavir-ritonavir, and remdesivir can lower glycemic control and raise mucormycosis^21^.

In addition to uncontrolled hyperglycemia, free unbound iron in the serum plays a role in the development of mucormycosis. As a result of acidosis and proton-mediated displacement of ferric iron from transferring, diabetic ketoacidosis, a key risk factor for mucormycosis, is linked to higher serum-free iron levels^24^.

COVID-19 is also a hyper-ferritinemic disorder. Serum ferritin levels that are too high result in the rise of intracellular iron, promoting hepatocyte apoptosis by creating reactive oxygen species. Cytokines, particularly IL-6, boost ferritin production while limiting iron export, trapping more iron inside the cell and exacerbating the situation^25^. Ferritin is released into the bloodstream after tissue damage; circulating ferritin loses some of its inner iron content, resulting in high free iron^26^. The development of ketoacidosis is another putative relationship between COVID-19 and mucormycosis. Even in the absence of diabetes mellitus, people with COVID-19 have developed ketonemia and ketoacidosis^27^. Acidosis reduces phagocytic function, which predisposes to mucormycosis and boosts free iron levels^20^.

The use of glucocorticoids is a known risk factor for developing mucormycosis^19^. Glucocorticoids produce immunosuppression, hyperglycemia, and lymphopenia, all of which predispose to the development of mucormycosis. The common usage of glucocorticoids in COVID-19 patients has contributed to an increase in C.A.M. cases^28–30^. Viral-induced lymphopenia and endothelities are two more variables that predispose COVID-19 patients to the development of C.A.M. Mucorales adherence and penetration to the endothelium may be aided by widespread endothelial damage reported in COVID-19 patients. Early phases in the pathophysiology of mucormycosis include endothelial adhesion and penetration^30^. Indeed to previous causes, early use of steroids in our patients had moderate to severe COVID-19 infection as a risk factor for catching mucormycosis.

As regards the severity of covid 19 as a predictor of M.A.C. as shown in a study by Riadetal., who found that the clinical symptoms and severity were reported in only 44.5% of the patients. Of those patients with available information, 40.8% were severe, 26.5% moderate, 18.4% mild, and 14.3% critical cases^31^, which correlate with our study.

The spread of fungal spores via water used in oxygen humidifiers is another indirect link between the concurrent rise in COVID-19 and mucormycosis. Indeed, hospital water can serve as a breeding ground for fungi like Mucorales^32^.

Furthermore, long-term usage of broad-spectrum antibiotics (carbapenems; averozolids) was a significant univariate risk factor. This is in line with the findings of 210-patient research on black fungus, which indicated that antibiotics were used to treat 100 percent of COVID-19 patients who were later diagnosed with mucormycosis. Antibiotics (Azithromycin, Doxycycline, and Carbapenems) enhance fungal infections in COVID patients in India.

In univariate analysis, high steroid doses were linked to black fungus infection, but this was not the case in multivariate analysis, which could be attributable to the early and prolonged use of steroids in all COVID 19 patients. According to the Randomized Evaluation of COVID-19 Therapy ('RECOVERY') Collaborative Group of the National Institute of Health, steroids should only be used in patients on a ventilator or require supplementary oxygen, not in milder cases^33^.

Furthermore, some of our participants could not afford treatment or had to discontinue using liposomal amphotericin due to financial difficulties. Participants who had combined medical and surgical care had a much better outcome, consistent with earlier research^34^. Surgical debridement of necrosed tissue likely allows antifungal medicines to penetrate more effectively, improving results. Those with rhino-orbital mucormycosis had the highest surgical rate. Unfortunately, even in the case of rhino-orbital illness, drastic surgery was not always possible. Patients with intracranial extension had a much higher mortality rate, with the majority being inoperable. Despite receiving proper antifungal treatment, death was significant among inoperable subjects, indicating the need for earlier detection and better therapeutic options.

Our research discovered various obstacles in controlling mucormycosis, including a delay in seeking medical help, a lack of expertise among clinicians, and financial constraints. In fact, despite being easily detectable by diagnostic sample, mucormycosis was detected after an extended period, which may explain the high fatality rate seen. Roden et al. investigated zygomycosis data in the English-language literature and assessed 929 eligible cases, finding that 96 percent of patients with disseminated illness died^35^.

In mucormycosis patients, the COVID-19 pandemic has spread. Over 6 months, retrospective observational research from an Egyptian tertiary care hospital discovered 12 rhino-orbito-cerebral mucormycosis (ROCM) (March 25, 2020, to September 25, 2020). During the same 6-month period in the preceding 3 years, the number of cases discovered was much higher than in the previous 3 years (1 case in 2017, 2 in 2018, and 1 in 2019)^36^.

Similarly, a prospective single-center observational study in India found 23 cases of invasive mucormycosis of the paranasal sinuses from August to December 2020^37^. This contrasts with a 2015 study conducted by two prominent medical institutes in Mumbai, India, which included only 20 cases over 3 years^38^. Song et al. published a study in April 2020 that looked into the link between Covid-19 and invasive fungal sinusitis, concluding that a large number of patients who have been exposed to or recovered from Covid-19 are at an increased risk of developing invasive fungal diseases and offering a treatment protocol for such cases^39^. Except for one patient who presented early with rhino orbital infection, most of our patients presented with rhino orbital cerebral mucormycosis, requiring late surgical intervention and costly antifungal treatment. This resulted in an inferior outcome, as more than 90% of the mucor patients studied died from multi-organ failure and septicemia. This explains why mucor patients showed higher inflammatory markers (E.S.R., D-dimer, C.R.P., A.K.I., and acute liver injury) and decreased platelets and lymphopenia.

Finally, we must reevaluate the logic and value of delivering steroids to COVID-19 patients regularly to avoid the rising number of iatrogenic deaths associated with steroid usage in people infected with SARS-CoV-2^40^.

## Limitation

In this cross-sectional retrospective analysis, first, clinical records revealed that the death rate is higher than in other studies due to late presentation and the legacy of expensive therapy. Secondly, more cohort multicenter larger studies are required to better assess the incidence and prevalence of C.A.M.

## Conclusions

Mucormycosis is a rare, severe and relatively fatal infection with neurological disabilities for survivors. It increased with COVID-19 due to immunity modulation and comorbidities, i.e., D.M., CKD, smoking. The main predisposing factor was uncontrolled diabetes mellitus, which was exacerbated by the use of glucocorticoids during the treatment of COVID-19. As a result, careful usage of this drug is required during the pandemic. Unfortunately, mucormycosis patients have a significant mortality rate even through adjunct surgery.

## Data Availability

All data generated or analyzed during this study are included in this published article.
